# Engaging and supporting national public health institutes as key climate actors: Amandine Ballart

**DOI:** 10.1093/eurpub/ckac129.224

**Published:** 2022-10-25

**Authors:** A Ballart, S Denys

**Affiliations:** 1 International Association of National Public Health Institutes, Paris, France; 2 Santé Publique France, Paris, France; 3 Global Health Institute, Emory University, Atlanta, USA

## Abstract

The International Association of National Public Health Institutes (IANPHI) collectively builds public health capacity by connecting, developing and strengthening national public health institutes worldwide. IANPHI recognizes climate action as a critical global public health intervention and acknowledges that public health interventions on environmental and socio-economic determinants of health are essential drivers of climate adaptation and mitigation. Recovery from the COVID-19 pandemic also provides a unique opportunity to reset business as usual and strengthen actions on climate change, biodiversity and health, while reducing social and health inequities in the long term. However, effective contributions of National Public Health Institutes (NPHIs) to adaptation plans and mitigation strategies for climate change have remained limited, with a primary focus on health surveillance to date. In this context, IANPHI developed a roadmap to support and reinforce the role of NPHIs in climate change mitigation and adaptation policies. Five priority actions were identified:

1. Advocate for strengthening NPHIs capacities to contribute effectively to climate and biodiversity research, policies, and action

2. Enhance capacity, competence and training through peer-to-peer support and knowledge sharing between NPHIs

3. Increase collaboration with international and regional organizations active in the fields of public health and climate change

4. Support the greening of public health services

5. Monitor progress in the NPHIs’ involvement in climate change policies through key indicators

In addition to discussing the roadmap, the presentation will focus on actions that IANPHI's Climate Change and Health committee initiated to reinforce the role of NPHIs in climate change mitigation and adaptation policies. This presentation will also provide information on how IANPHI can engage and support national public health institutes as key actors in the fight against climate change.

